# A Novel Formulation of Tigecycline Has Enhanced Stability and Sustained Antibacterial and Antileukemic Activity

**DOI:** 10.1371/journal.pone.0095281

**Published:** 2014-05-28

**Authors:** Yulia Jitkova, Marcela Gronda, Rose Hurren, Xiaoming Wang, Carolyn A. Goard, Bozhena Jhas, Aaron D. Schimmer

**Affiliations:** Princess Margaret Cancer Centre, University Health Network, Toronto, Canada; Università degli Studi di Firenze, Italy

## Abstract

Tigecycline is a broad-spectrum, first-in-class glycylcycline antibiotic currently used to treat complicated skin and intra-abdominal infections, as well as community-acquired pneumonia. In addition, we have demonstrated that tigecycline also has *in vitro* and *in vivo* activity against acute myeloid leukemia (AML) due to its ability to inhibit mitochondrial translation. Tigecycline is relatively unstable after reconstitution, and this instability may limit the use of the drug in ambulatory infusions for the treatment of infection and may prevent the development of optimal dosing schedules for the treatment of AML. This study sought to identify a formulation that improved the stability of the drug after reconstitution and maintained its antimicrobial and antileukemic activity. A panel of chemical additives was tested to identify excipients that enhanced the stability of tigecycline in solution at room temperature for up to one week. We identified a novel formulation containing the oxygen-reducing agents ascorbic acid (3 mg/mL) and pyruvate (60 mg/mL), in saline solution, pH 7.0, in which tigecycline (1 mg/mL) remained intact when protected from light for at least 7 days. This formulation also preserved the drug's antibacterial and antileukemic activity *in vitro*. Moreover, the novel formulation retained tigecycline's antileukemic activity *in vivo*. Thus, we identified and characterized a novel formulation for tigecycline that preserves its stability and efficacy after reconstitution.

## Introduction

Tigecycline is a first-in-class glycylcycline antibiotic, developed as a third generation structural analog of older tetracyclines [1], [2]. It displays broad-spectrum, potent activity against both Gram-positive and Gram-negative bacteria, including many multidrug-resistant pathogens [2], [3]. Tigecycline is currently approved in North America for the treatment of adults with complicated skin, skin structure or intra-abdominal infections, or those with community-acquired pneumonia [4]. The drug has also been used for the treatment of additional infections, including nosocomial sepsis, bacteremia and ventilator-associated pneumonia [5].

Beyond its role as an antimicrobial, we recently identified tigecycline as an agent with novel anticancer activity in preclinical studies of human acute myeloid leukemia (AML) [6]. Tigecycline was preferentially cytotoxic to AML cells, including leukemic stem and progenitor cells, compared to normal hematopoietic cells *in vitro* and *in vivo* [6]. In addition, similar sensitivity to tigecycline was observed across all cytogenetic risk groups. Thus, tigecycline may have clinical activity beyond its role as an antimicrobial agent.

Mechanistically, the antibacterial activity of tigecycline is attributable to strong binding of the drug to the 30S subunit of the bacterial ribosome, preventing peptide elongation and thereby disrupting protein translation [7], [8], [9]. Stacking interactions between the unique 9-t-butylglycylamido group of tigecycline and the 16S rRNA of the 30S ribosome subunit enhance the binding affinity and antibacterial potency of this drug compared to other tetracycline antibiotics [9]. Moreover, the bulkiness of this moiety circumvents the common mechanisms of tetracycline resistance [7], [9]. Interestingly, the molecular mechanism underlying the antileukemic effects of tigecycline in human cells also involves inhibition of protein translation, in this case, in the mitochondria [6]. In mammalian cells, mitochondrial ribosomes support the synthesis of 13 proteins encoded by the mitochondrial genome, which assemble with imported nuclear-encoded proteins to form a functional respiratory chain for oxidative phosphorylation [10]. Given that mitochondrial biogenesis and energetics appear to be dysregulated in AML cells [6], [11], [12], the pharmacological disruption of mitochondrial translation may have potential as a novel antileukemic therapeutic strategy with a promising therapeutic window [6], [13].

A challenge in the clinical administration of tigecycline is its poor stability. The phenol group in tigecycline leaves it susceptible to oxidation, particularly at pH values greater than 7 [14], [15], [16]. At lower pH, tigecycline is more prone to nonenzymatic epimerization [14]. Both of these chemical processes result in pharmacologically inactive products. For clinical use, tigecycline is currently formulated as a lyophilized powder or cake, which is reconstituted (10 mg/mL) and diluted (1 mg/mL) for intravenous administration [4]. The marketed formulation of tigecycline (Tygacil) includes the excipients lactose monohydrate to stabilize the drug against epimerization, and hydrochloric acid/sodium hydroxide to adjust the pH to prevent oxidation [4], . Even with these stabilizing additives present, however, tigecycline can only be stored for 6 h at room temperature following reconstitution, and for an additional 18 h once diluted in an intravenous bag at room temperature [4].

The poor stability of tigecycline after reconstitution largely precludes the use of this drug for the treatment of infection on an ambulatory basis by home infusion as the infusion bags would have to be changed very frequently. In addition, the poor stability also limits the study of its potential antileukemic activity, as ambulatory continuous infusion schedules cannot be readily evaluated. For these reasons, we have developed a formulation of tigecycline that preserves the stability of the drug after reconstitution for up to 7 days and maintains its antibacterial and antileukemic activity.

## Materials and Methods

### Materials

Tigecycline powder was obtained from Sequoia Research Products (Pangbourne, United Kingdom). Oxyrase was purchased from Oxyrase, Inc. (Mansfield, OH). Hyclone fetal calf serum was from Thermo Fisher Scientific (Rockford, IL). Interleukin 3 (IL-3) and stem cell factor (SCF) were from R&D Systems (Burlington, ON, Canada), and L-glutamine was from Life Technologies (Burlington, ON, Canada). Mueller Hinton Broth was obtained from BD (Franklin Lakes, NJ), Iscove's modified Dulbecco's medium (IMDM) was prepared from powder (Cat # 12200, Invitrogen (Burlington, ON, Canada)) at the Ontario Cancer Institute Tissue Culture Media Facility (Toronto, ON, Canada). All other reagents were purchased from Sigma unless otherwise noted.

### HPLC Tigecycline stability assay

#### Preparation of tigecycline

Tigecycline stock solution (25 mg/mL) was made in saline, pH 7. Tigecycline was then diluted to 1–5 mg/mL in saline containing different additives as indicated in figure legends, adjusted to pH 7 and incubated at room temperature. For incubations in the dark, sample tubes were wrapped in aluminium foil. Just before initiating the assay, samples were diluted with the mobile phase to 70–350 µg/mL and analyzed by high performance liquid chromatography (HPLC).

#### Preparation of standards and samples

Tigecycline standards were prepared by dilution of the stock solution in saline in the range of 7.0–5600 µg/mL. Five microliters of all tigecycline standards and samples was diluted with 65 µL mobile phase (4 mM 1-octanesulfonic acid in acetonitrile:monosodium phosphate, pH 3 (25∶75, v/v)) and subjected to HPLC detection of tigecycline.

#### HPLC analysis

Samples, quality controls and tigecycline standards were subjected to HPLC with UV detection using a Shimadzu LC-10AD equipped with a Symmetry C18 column (Waters; 3.9×150 mm, 100 Å pore size, 5 µm particle size). The mobile phase was 4 mM 1-octanesulfonic acid in (25∶75, v/v) acetonitrile:monosodium phosphate, (0.023 M, pH 3.0), the flow rate was set to 1.2 mL/min, and ultraviolet detection was performed at 244 or 350 nm as previously described [17]. The retention time of intact tigecycline was 5.6 min. Tigecycline stability was calculated based on the area under the peak of intact tigecycline, relative to a reference detected from tigecycline freshly dissolved in saline at an equal concentration. Plasma concentrations of tigecycline were calculated based on a calibration curve using tigecycline standards.

### Antibacterial activity assay

The minimal inhibitory concentration (MIC) of tigecycline in *E. coli* (strain MG1655; a kind gift from Dr. Shana O. Kelley, University of Toronto, Toronto, ON, Canada) was determined using a published protocol [18] with some modifications. Media was treated with Oxyrase to remove oxygen and therefore stabilize tigecycline for 16–20 hours of the assay, as described [15]. The working concentration of tigecycline was 2–1000 ng/mL and the working stock of bacteria was 10^6^ cfu/mL. Tigecycline and samples were protected from light throughout the experiment.

### Human leukemic cell culture

Human leukemic OCI-AML2 [19], HL-60 (ATCC) and TEX cell lines [20] were obtained as a kind gift from Dr. Minden, Dr. Kamel-Reid and Dr. Dick, respectively (Princess Margaret Cancer Centre, Toronto, ON, Canada) and were maintained in Iscove's modified Dulbecco's medium (IMDM). All media were obtained from the Ontario Cancer Institute Tissue Culture Media Facility (Toronto, ON, Canada) and supplemented with 10% fetal calf serum (FCS), 100** µ**g/mL penicillin and 100** µ**g/mL streptomycin (Hyclone, Logan, UT), except for TEX. TEX cell medium was supplemented with 15% fetal calf serum (FCS), 2 mM L-glutamine, 100** µ**g/mL penicillin, 100** µ**g/mL streptomycin, 20 ng/mL SCF, and 2 ng/mL IL-3. Cells were incubated at 37°C in a humidified air atmosphere supplemented with 5% carbon dioxide (CO_2_).

### Cell viability assays

Cell growth and viability were measured with CellTiter-Fluor (Promega) or AlamarBlue (Life Technologies) viability assays according to the manufacturers' instructions. For CellTiter-Fluor assays, stock solutions of tigecycline in saline with or without additives were preincubated at room temperature for 4 days, and cells were treated with 0.63–30 µM tigecycline for 72 h before the assay. Alamar Blue assays, tigecycline stocks were similarly preincubated for 5 days, and TEX cells were similarly treated for 48 h. Tigecycline and samples were protected from light throughout the experiment.

### Immunoblotting

Total cell lysates were prepared from cells as described previously [21]. Briefly, cells were washed twice with phosphate buffered saline pH 7.4 and suspended in lysis buffer (1.5% n-dodecyl β-maltoside (Sigma Aldrich, St. Louis, MO)) containing protease inhibitor tablets (Complete tablets; Roche, IN). Protein concentrations were measured by the DC Protein assay (Bio Rad, Hercules, CA). Equal amounts of protein were subjected to sodium dodecyl sulphate (SDS)-polyacrylamide gels followed by transfer to PVDF membranes. Membranes were stained with 0.1% Amido Black in 10% acetic acid for 2 minutes. Membranes were then probed with anti-Cox-1 1**∶**2000 (Santa Cruz Biotechnology Inc), anti-Cox-2 1**∶**1000 (Abcam, Cambridge, UK), anti-Cox-4 1**∶**8000 (Molecular Probes) and secondary antibodies from GE Health (IgG peroxidase linked species-specific whole antibody). Detection was performed by the enhanced chemical luminescence method (Pierce, Rockford, IL).

### Complex IV (cytochrome c oxidase) activity assay

To evaluate mitochondrial respiratory complex IV activity upon tigecycline treatment, TEX cells were treated for 48 hours with 5 µM tigecycline either freshly dissolved in saline with or without additives or following a 4-day incubation in the dark. Then mitochondria were isolated and analyzed for protein content, citrate synthase activity and complex IV activity.

Intact mitochondria were isolated from TEX cells as described by Frezza *et al*. [22], with minor modifications. Confluent cells were harvested and frozen on dry ice. Next, cell pellets were thawed on ice and lysed by hypotonic shock in distilled water for 10 min at 4°C. Lysates were then transferred to ice cold buffer A (250 mM sucrose; 10 mM Tris-HCl, pH 7.0; and 0.2 mM EGTA, pH 8.0) and spun at 600 g at 4°C for 10 min. Supernatants were collected and centrifuged at 12,000 g for 12 min at 4°C. Supernatants were then discarded and pellets were washed twice in ice-cold buffer A. Pellets, containing mitochondria, were frozen in dry ice and then transferred to a −80°C freezer for storage until use. Mitochondrial protein concentration was determined using the Bradford protein assay kit (BioRad).

Complex IV activity was measured by KCN-sensitive oxidation of ferrocytochrome c and normalized to citrate synthase activity as an indicator of mitochondrial mass, as described in our previous report [6].

### Pharmacokinetic studies

NOD/SCID mice were administered 50 mg/kg tigecycline or novel formulation of tigecycline by intraperitoneal injection (n = 2 mice per control group; n = 3 mice per treatment group). Venous blood samples were collected at 15, 30, 60, 120, 240 and 360 minutes after drug injection. Tigecycline was assayed in 22 µL mouse plasma by HPLC with UV detection (350 nm) from 0.9 to 120 µg/mL. Plasma was diluted with 56** µ**L PBS to improve tigecycline extraction from plasma, and proteins were precipitated by addition of 17.5** µ**L of acetonitrile and 8** µ**L 100%-trichloroacetic acid. Plasma samples were centrifuged at 13000 rpm for 15 min at 4°C, and then the aqueous phase was loaded on the HPLC column. The calibration curve was built using mouse plasma spiked with 1.7 to 58.5 µg/mL tigecycline in PBS. The peak plasma concentration (*C*_max_), the terminal half-life (*t*_1/2_), area under the plasma concentration-time curve (AUC), clearance (CL) and volume of distribution (*V*z) were evaluated using WinNonlin version 6.2.1 (Pharsight).

### Antileukemic activity assessment of the novel formulation in mouse model of human leukemia

OCI-AML2 human leukemia cells (5×10^5^) were injected subcutaneously into the flanks of sub-lethally irradiated (3.5 Gy) NOD/SCID mice (Ontario Cancer Institute, Toronto, ON). 11 days after injection, once tumors were palpable, mice were treated with 50 mg/kg of tigecycline, novel formulation of tigecycline, or vehicle controls (saline or formulation) by intraperitoneal injection twice a day for 11 days (n = 9 per group). After 11 days of treatment, mice were sacrificed, tumors excised and tumor weight measured. Animal studies were performed with approval from the Ontario Cancer Institute/Princess Margaret Cancer Centre Animal Care Committee.

### Toxicity assessment of the novel formulation in mice

NOD/SCID mice were treated with the novel formulation solvent (60 mg/mL pyruvate, 3 mg/mL ascorbic acid, pH 7 in saline) by intraperitoneal injection once daily, 5 times per week (at least 3 mice per treatment group). After 3 weeks of treatment, the animals were sacrificed, and organs were embedded with 10% buffered formalin, sectioned and analyzed, and serum was analyzed for bilirubin, aspartate phosphatase, alkaline phosphatase, and creatine kinase levels. Mouse experiments were performed with approval from the Ontario Cancer Institute/Princess Margaret Cancer Centre Animal Care Committee.

## Results

### Identification of excipients that stabilize tigecycline in solution

**Figure 1:**
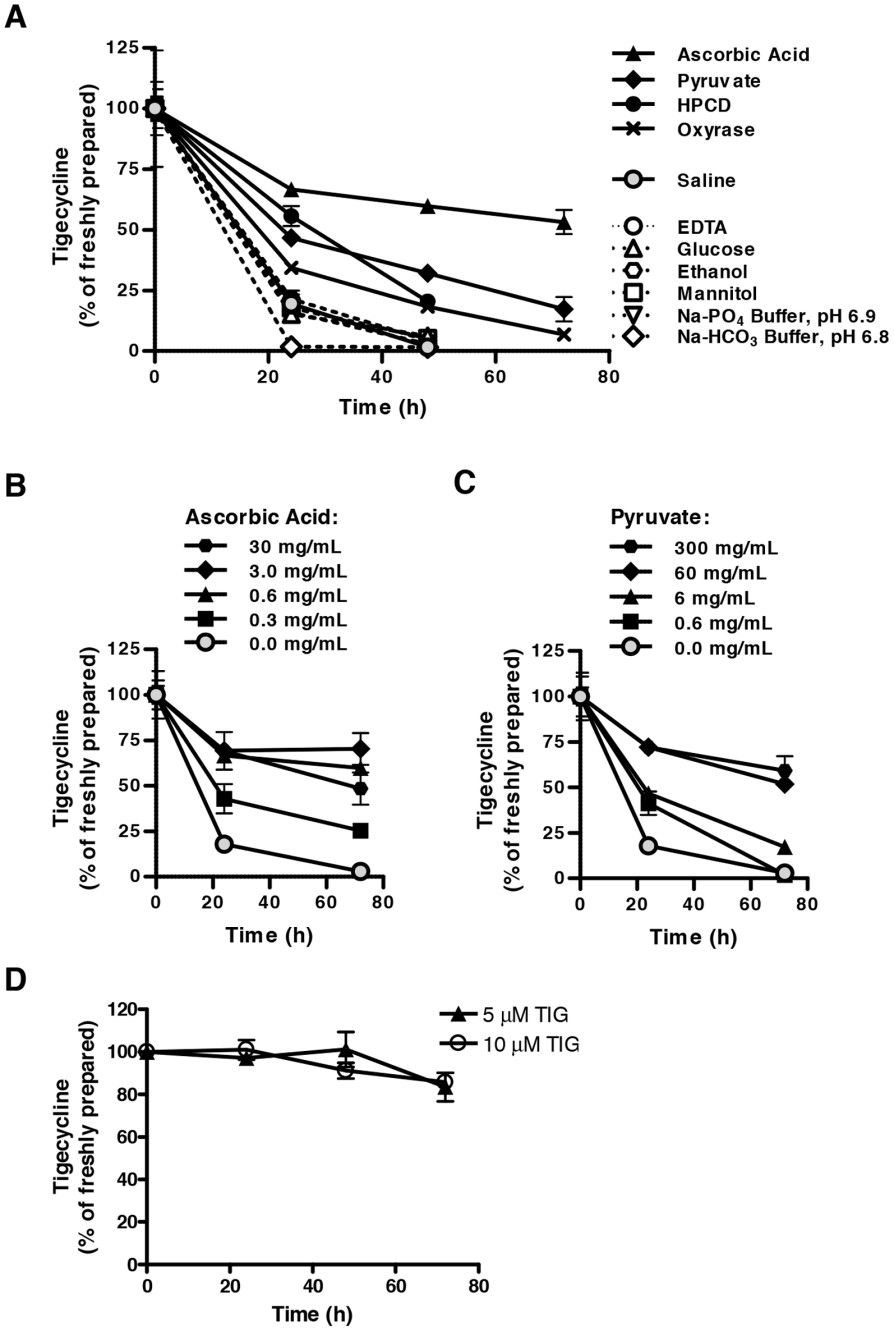
Ascorbic acid and pyruvate stabilize tigecycline in saline solution. Tigecyline (1 mg/mL) was dissolved in saline solution with or without supplementation with various excipients and incubated in the light. Tigecycline concentrations were detected over time by HPLC and expressed as a relative percentage of that detected immediately following fresh dilution in saline ( = 100%). Tigecycline was dissolved in (**A**) saline with or without 0.6 mg/mL ascorbic acid, 6 mg/mL pyruvate, 50 mg/mL 2-hydroxypropyl-β-cyclodextrin (HPCD), 0.3 U/mL Oxyrase with 20 mM sodium lactate, 6 mg/mL EDTA sodium, 5% (w/v) glucose, 5% (w/v) mannitol, 10% (v/v) ethanol, 10 mM sodium phosphate (Na-PO_4_) buffer pH 6.9, or 10 mM sodium bicarbonate (Na-HCO_3_) buffer pH 6.8; (**B**) saline containing 0–30 mg/mL ascorbic acid; or (**C**) saline containing 0–300 mg/mL pyruvate; (**D**) Iscove's modified Dulbecco's medium (IMDM). In all panels, data indicate the mean ± standard deviation of 3 independent experiments.

To improve the stability of tigecycline after reconstitution, we investigated the effect of 11 chemical additives on the stability of 1 mg/mL tigecycline in saline solution. These additives were selected to cover classes of excipients including: pH-modifying agents or buffers (sodium phosphate and sodium bicarbonate buffers); sugars, sugar alcohols, or polysaccharides (glucose, mannitol, and 2-hydroxypropyl-β-cyclodextrin (HPCD)); edetate calcium chelators (EDTA); and antioxidant reagents (L-ascorbic acid, sodium pyruvate, and Oxyrase). In addition, we assessed the stability of tigecycline in a 10% (v/v) solution of ethanol in saline solution to evaluate the feasibility of formulating the drug in an alcohol-containing suspension for oral administration. Tigecycline in saline with or without single additives was incubated at ambient room temperature for up to 3 days, with no precautions taken to protect the solution from light. The stability of tigecycline was assessed by HPLC (**Figure 1A** **,** **Tables 1** **,** **2**). When maintained in saline alone, tigecycline rapidly degraded. Only 20% of dissolved tigecycline remained intact after 24 hours, and less than 2% remained intact after 48 hours. In contrast, as single additives, all three antioxidant reagents enhanced tigecycline stability. After 48 hours, 18%, 32%, and 68% of the drug remained intact in solutions supplemented with Oxyrase, pyruvate, and ascorbic acid, respectively. HPCD also enhanced tigecycline stability, whereby 20% of the drug remained intact after 48 hours. All other additives appeared to have a negligible impact on tigecycline stability, or in the case of the sodium bicarbonate buffer, even a destabilizing effect. Ascorbic acid and pyruvate demonstrated the greatest stabilization of tigecycline, and this effect was dose dependent up to approximately 3 mg/mL ascorbic acid and 60 mg/mL pyruvate (**Figure 1B–C**).

**Table 1:** Activity of tigecycline (1 mg/mL) when freshly prepared in saline.

Composition (mg/mL)				Tigecycline by HPLC (% of freshly prepared)	Bacterial MIC (ng/mL)*	CellTiter IC_50_ in TEX cells (μM)*	Alamar blue IC_50_ in TEX cells (μM)†	Complex IV activity in TEX cells (% of control)†
Tig	Pyr	CD	AA					
1.0	—	—	—	100.0±1.8	125	4.56±0.27	4.33±0.13	39.2±14.9

*Tigecycline solution was preincubated at room temperature for 4 days, which was then used to determine the minimum inhibitory concentration (MIC) in *Escherichia coli* (MG1655) and mean half-maximal inhibitory concentration (IC_50_) in TEX cells using the CellTiter viability assay following incubations with bacteria (24 hours) or cells (72 hours), respectively.

† Tigecycline solution was preincubated at room temperature for 5 days, and TEX cells were then incubated with 5** µ**M tigecycline for 48 hours. The IC_50_ for cell viability was then assessed by the Alamar blue assay, and respiratory complex IV activity was determined in cell lysates. Activity was normalized to citrate synthase content as a proxy for mitochondrial mass.

All solutions were adjusted to pH 7 and all incubations were performed in the dark. Numbers indicate mean ± standard deviation of at least 3 independent experiments.

Tig, tigecycline; Pyr, pyruvate; CD, 2-hydroxypropyl-β-cyclodextrin; AA, ascorbic acid.

**Table 2:** Stability and activity of tigecycline reconstituted in saline with or without additives.

Composition (mg/mL)				Tigecycline by HPLC (% of freshly prepared)						Bacterial MIC (ng/mL)*	CellTiter IC_50_ in TEX cells (μM)*	Alamar blue IC_50_ in TEX cells (μM)*	Complex IV activity in TEX cells (% of control)†
Tig	Pyr	CD	AA	Preincubation (days)						Preincubation (days)			
				0 (fresh)	3	4	5	6	7	4	4	5	5
1.0	—	—	—	100.0±10.0	12.2±0.7	15.1±3.2	8.4±2.5	10.2±2.9		>2000	>50.00	>50.00	100.0±6.4
2.5	—	—	—	100.0±25.0	2.7±0.7								
5.0	—	—	—	100.0±4.2	1.9±0.3								
1.0	60.0	—	—	100.0±12.1	61.2±8.4			42.2±0.2					
1.0	—	50.0	—	100.0±5.8	33.7±10.8			16.2±0.3					
1.0	—	—	0.3	100.0±5.8	30.2±5.7			25.7±5.9					
1.0	—	—	0.6	100.0±13.3	54.4±7.9			46.6±1.5	32.1				
1.0	—	—	1.0	100.0±2.6	78.9±6.2				53.2				
1.0	—	—	2.0	100.0±4.6	62.6±1.1				53.2				
1.0	—	—	3.0	100.0±10.0	67.6±5.6		50.6		56.7				
1.0	60.0	—	0.3	100.0±5.6	85.5±14.4			60.4±1. 1	45.5±6.5				
1.0	60.0	—	0.6	100.0±10.6	106.5±6.7	87.5±8.4	86.8±10.8	86.8±3.6	76.1±3.2				
1.0	60.0	—	1.0	100.0±3.9	95.5±5.1	92.8±2.7	93.6±2.6		77.4±2.6	125	4.22±0.30		35.1±7.7
1.0	60.0	—	2.0	100.0±8.7	101.7±10.8	97.0±7.5	96.0±2.8		89.7±8.7	125	4.74±0.68	4.76±0.28	37.8±11.5
1.0	60.0	—	3.0	100.0±7.1	101.2±4.1	105.0±7.8	99.7±2.3		92.5±6.1	125	5.20±0.59	4.25±0.26	44.3±10.7
2.5	60.0	—	3.0	100.0±18.0	114.8±3.1	96.4±15.7	87.1±18.1		59.6±12.0		5.00±0.73		
5.0	60.0	—	3.0	100.0±2.3	77.3±5.6	61.9±9.3	48.0±4.8		26.4±1.5				
1.0	—	50.0	0.3	100.0±7.5	51.1±7.3			35.7±5.1					
1.0	—	50.0	0.6	100.0±11.4	65.4	56.7	48.2		48.1				
1.0	—	50.0	1.0	100.0±4.4	84.3±6.25	71.4±2.9	65.2±1.8		55.2±5.9				
1.0	—	50.0	2.0	100.0±0.9	69.9±4.1	58.1±3.8	56.2±5.2		48.0±22.0				
1.0	—	50.0	3.0	100.0±3.3	53.0±8.75	66.5±10.7	44.7±8.8		44.0±29.0				
1.0	60.0	50.0	—	100.0±7.1	82.9±9.3			67.5±8.1	58.6±2.2				
1.0	60.0	50.0	0.3	100.0±10.2	72.2±8.6			67.4±5.0					
1.0	60.0	50.0	0.6	100.0±13.2	90.9±4.0	74.8±6.8	89.0±0.3	75.2±11.9	76.5±0.2				
1.0	60.0	50.0	1.0	100.0±1.12	94.9±9.0	98.5±7.5	94.0±8.1		80.5±7.8	125	4.50±0.60		31.6±11.0
1.0	60.0	50.0	2.0	100.0±1.13	97.5±7.1	99.6±3.5	93.6±3.1		83.0±8.2	125	4.16±0.51	4.68±0.28	40.1±11.9
1.0	60.0	50.0	3.0	100.0±4.04	97.4±2.6	99.9±3.1	104.5±0.6		88.4±8.1	125	4.62±0.66	4.32±0.28	33.2±3.5
2.5	60.0	50.0	0.3	100.0±15.2	74.1±1.1			51.8±5.8					
2.5	60.0	50.0	0.6	100.0±1.4	78.0±0.1			56.0±13.1					
2.5	60.0	50.0	2.0	100.0±15.2	90.8±13.1	93.7±6.1	87.1±11.1		68.0				
2.5	60.0	50.0	3.0	100.0±1.4	87.1±22.9	88.9±22.5	81.0±18.8		63.0±17.5				

*Tigecycline solution was preincubated at room temperature for 4 days, which was then used to determine the minimum inhibitory concentration (MIC) in *Escherichia coli* (MG1655) and mean half-maximal inhibitory concentration (IC_50_) in TEX cells using the CellTiter viability assay following incubations with bacteria (24 hours) or cells (72 hours), respectively.

† Tigecycline solution was preincubated at room temperature for 5 days, and TEX cells were then incubated with 5** µ**M tigecycline for 48 hours. The IC_50_ for cell viability was then assessed by the Alamar blue assay, and respiratory complex IV activity was determined in cell lysates. Activity was normalized to citrate synthase content as a proxy for mitochondrial mass.

All solutions were adjusted to pH 7 and all incubations were performed in the dark. Numbers indicate mean ± standard deviation of at least 3 independent experiments, where applicable.

Tig, tigecycline; Pyr, pyruvate; CD, 2-hydroxypropyl-β-cyclodextrin; AA, ascorbic acid.

Of note, when dissolved in IMDM medium that contains pyruvate, tigecycline was stable for up to 72 hours at concentrations of 5 and 10** µ**M (**Figure 1D**).

### Tigecycline stability is enhanced in a novel solution formulation, protected from light

**Figure 2:**
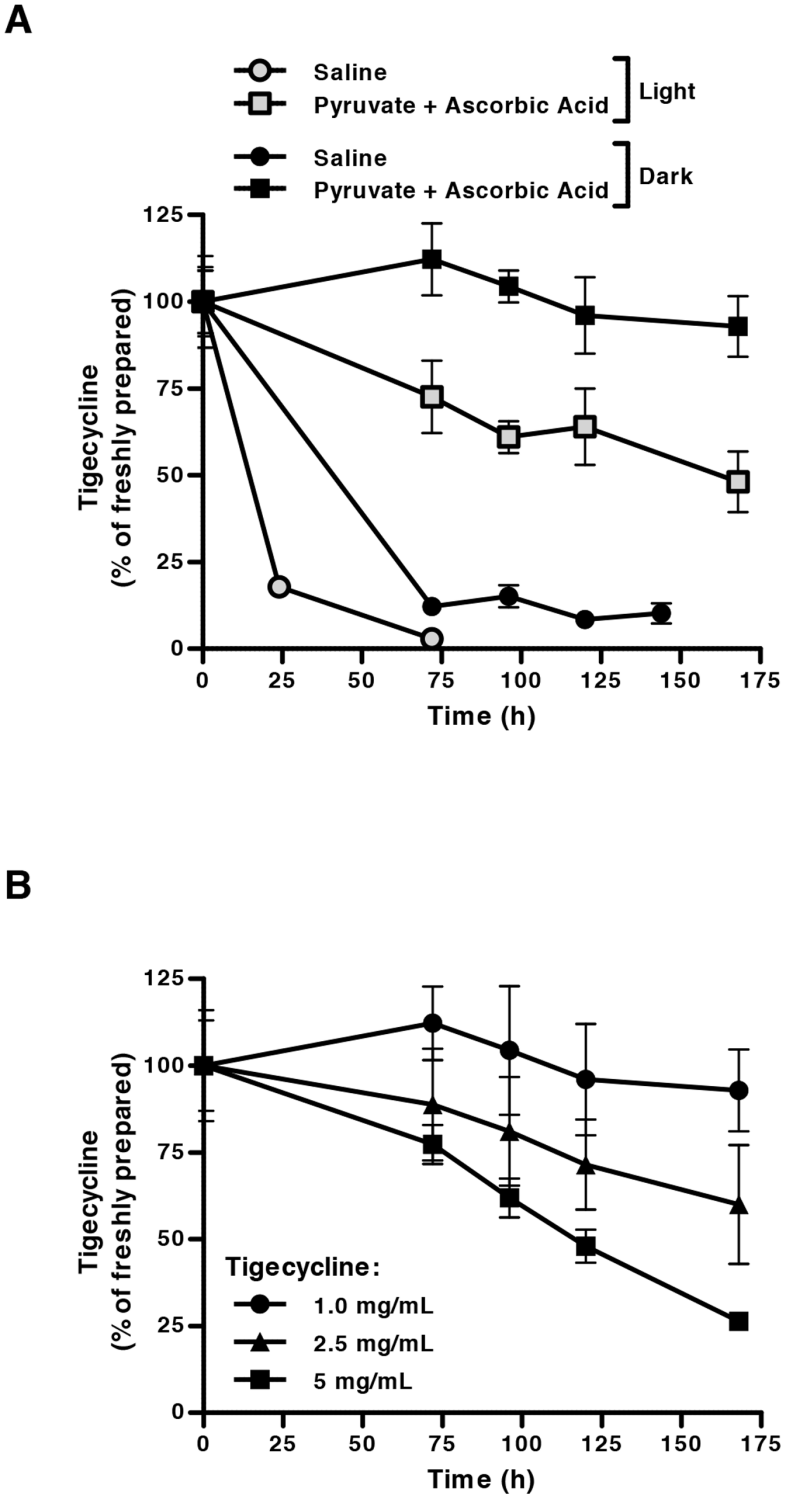
Tigecycline is stabilized in solution under novel formulation conditions for up to 7 days. Following dissolution in saline or a novel stabilizing formulation containing ascorbic acid (3 mg/mL) and pyruvate (60 mg/mL) in saline, adjusted to pH 7, tigecycline concentrations were detected by HPLC and expressed as a relative percentage of that detected following fresh dilution in saline ( = 100%). (**A**) Tigecycline (1 mg/mL) dissolved in saline or the novel formulation was incubated under light or dark conditions, demonstrating light sensitivity. (**B**) Increasing concentrations of tigecycline were dissolved in the novel formulation and incubated in the dark. Tigecycline stability in solution was reduced at concentrations greater than 1 mg/mL. In all panels, data indicate the mean ± standard deviation of 3 independent experiments.

Given the encouraging effects of ascorbic acid and pyruvate as single agents on tigecycline stability, we evaluated a novel formulation solvent for the drug containing 3 mg/mL ascorbic acid and 60 mg/mL pyruvate in saline solution (pH 7.0). This combination of additives improved the stability of tigecycline (**Figure 2A**) to a greater extent than either additive alone (**Figure 1**). Moreover, 72 hours after incubation, the combination of ascorbic acid and pyruvate in saline increased the stability of tigecycline 25-fold in comparison to when tigecycline was reconstituted in saline alone (**Figure 2A**). By 7 days after reconstitution in the novel formulation, almost 50% of tigecycline remained (**Figure 2A**).

We also evaluated the impact of light exposure on the stability of tigecycline after reconstitution. When incubated in the novel formulation and protected from light, tigecycline was even more stable, maintaining greater than 90% integrity over 7 days (**Figure 2A**). We therefore identified supplementation with ascorbic acid and pyruvate and protection from light as promising tigecycline formulation conditions. Of note, we also evaluated the impact of including HPCD in the stabilizing formulation. The addition of HPCD did not enhance the stabilization of tigecycline compared to ascorbic acid and pyruvate alone (data not shown).

We next evaluated whether the stability of tigecycline varied with the concentration of tigecycline itself. A comprehensive analysis of the impact of different concentrations of tigecycline and combinations of additives on the stability of different concentrations of tigecycline over time is displayed in **Tables 1** **and** **2**. As the concentration of tigecycline in solution increased, its stability after reconstitution decreased (**Figure 2B**). We also calculated the rate of degradation of tigecycline as a function of its concentration. After reconstitution in either saline or ascorbic acid and pyruvate, the rate of tigecycline degradation over time displayed a positive linear relationship with tigecycline concentration, consistent with first-order degradation reaction kinetics. Linear regression demonstrated that the rate constant of tigecycline degradation in saline (k = 0.335 day^−1^, R^2^ = 1.000) was greater than that in the novel formulation (k = 0.132 day^−1^, R^2^ = 0.984). This indicates that the rate of tigecycline degradation was 2.5-fold slower when reconstituted in the ascorbic acid- and pyruvate-containing formulation, compared to when reconstituted in saline alone.

### Tigecycline reconstituted in ascorbic acid and pyruvate maintains its antibacterial and antileukemic activities

Since the novel antioxidant-containing formulation of tigecycline improved its stability for up to one week, we tested whether its antibacterial and antileukemic activities would be maintained after reconstitution in ascorbic acid- and pyruvate-supplemented saline (**Tables 1** **and** **2**). The mean inhibitory concentration (MIC) of tigecycline freshly prepared in saline for *E. coli* (MG1655 strain) was approximately 125 ng/mL. However, when this solution was incubated in the dark at room temperature for 4 days, tigecycline degraded and lost its antibacterial efficacy with an MIC >2** µ**g/mL. In contrast, if tigecycline was reconstituted in ascorbic acid and pyruvate, its full antibacterial activity was maintained following an identical 7 day preincubation (MIC  = 125 ng/mL). Similarly, after a 4 day preincubation of tigecycline in saline, tigecycline lost its ability to kill TEX human leukemia cells (from IC_50_∼5** µ**M when freshly prepared to IC_50_>50** µ**M after 4 days preincubation) as measured by CellTiter Flour assay. However, reconstitution of tigecycline in ascorbic acid and pyruvate preserved the anti-leukemic activity after the same 4 day preincubation when tested in TEX, OCI-AML2 and HL60 leukemia cells (**Tables 2** **,** **3**).

**Table 3:** Antileukemic activity of tigecycline reconstituted in saline with pyruvate and ascorbic acid.

Composition (mg/mL)		CellTiter	
Pyr	AA	IC_50_ in AML2 cells (μM)*	IC_50_ in HL60 cells (μM)*
**Preincubation, days**		**fresh**	
—	—	4.72±0.54	3.06±0.85
**Preincubation, days**		**4**	**4**
60	1	5.64±0.55	4.27±0.45
60	2	5.02±0.60	4.39±0.44
60	3	4.09±0.41	3.95±0.39

*Tigecycline solution (1 mg/mL) was preincubated at room temperature for 4 days, which was then used to determine the mean half-maximal inhibitory concentration (IC_50_) in OCI-AML2 and HL60 cells using the CellTiter viability assay following 72 hour incubation.

Pyr, pyruvate; AA, ascorbic acid.

We previously demonstrated that the disruption of mitochondrial translation in tigecycline-treated leukemic cells is accompanied by a decrease in the enzymatic activity of respiratory complex IV, which contains several mitochondrially-translated subunits [6]. This activity was lost when tigecycline was reconstituted in saline and incubated at room temperature for 5 days, but maintained when tigecycline was reconstituted in ascorbic acid and pyruvate (**Tables 1** **and** **2**).

### The novel stabilizing formulation of tigecycline maintains antileukemic activity in mouse model of human leukemia

**Figure 3:**
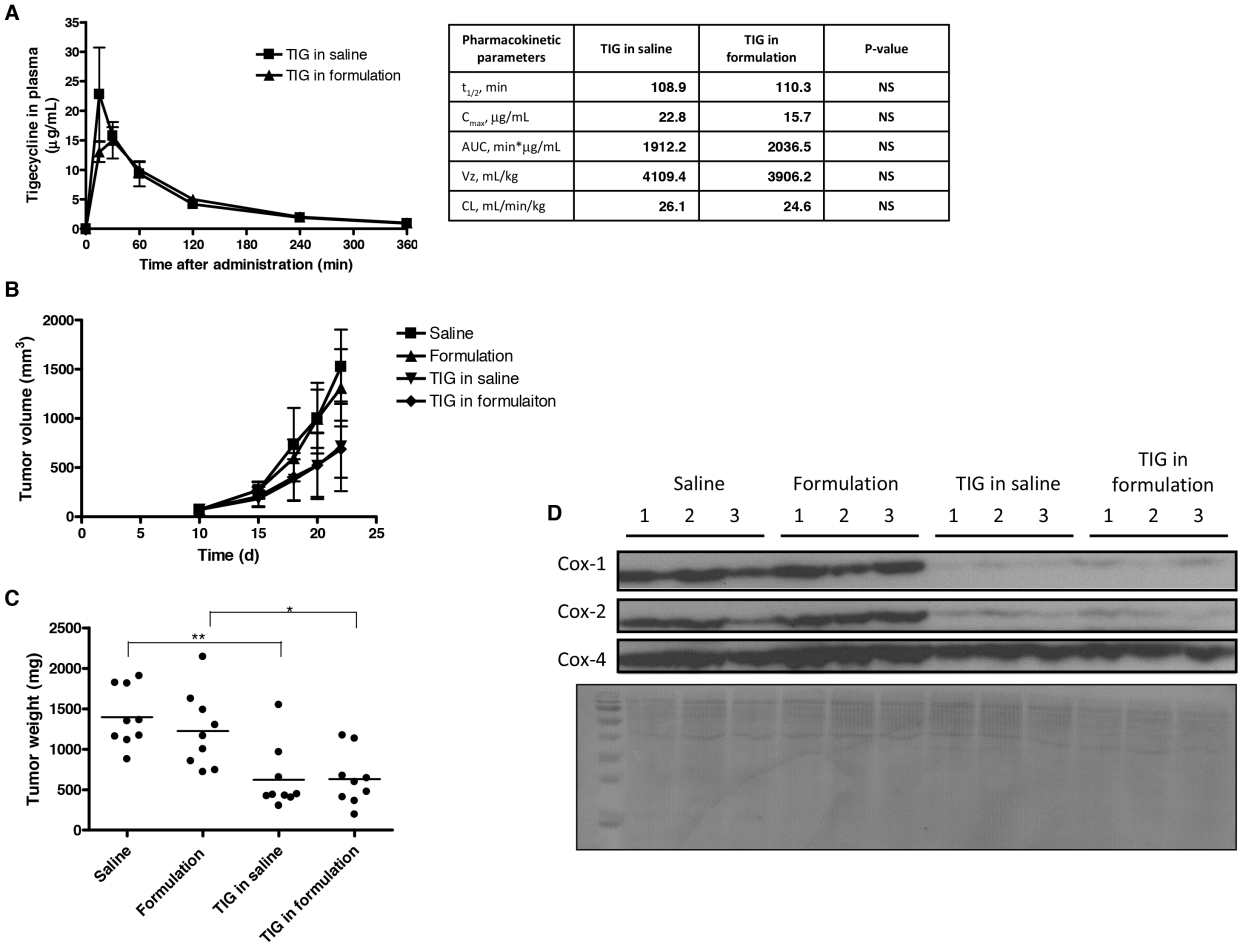
The novel ascorbic acid- and pyruvate-containing formulation displays efficacy in AML cells grown *in vivo*. **(A)** Mice were administered 50/kg tigecycline or 50 mg/kg novel tigecycline formulation by intraperitoneal injection and plasma was collected at increasing times after treatment. Plasma tigecycline concentration was determined using HPLC. The peak plasma concentration (*C*_max_), the terminal half-life (*t*_1/2_), area under the plasma concentration-time curve (AUC), clearance (CL) and volume of distribution (*V*z) were evaluated using WinNonlin 6.2.1. Data represent the mean ± standard deviation of a representative experiment with 3 mice per group. Human leukemia OCI-AML2 cells were injected subcutaneously into the flank of NOD/SCID mice. Eleven days after injection, once tumors were palpable, mice were treated with 50 mg/kg of tigecycline, novel formulation of tigecycline, or vehicle controls (saline or formulation) by intraperitoneal injection twice a day for 11 days (n = 9 per group). Tigecycline in each formulation was prepared fresh twice a day. **(B)** Tumor volume was monitored over time. Eleven days after injection, mice were sacrificed and tumors excised. **(C)** Tumor weight was measured. ** indicates p<0.01 and * indicates p<0.05 as determined by Tukey's post-test and one-way ANOVA analysis. Lines represent median. **(D)** Total proteins were extracted and analyzed by immunoblotting for Cox-1, Cox-2 and Cox-4 expression. PVDF membrane was stained with 0.1% Amido Black.

Given the encouraging stabilization of reconstituted tigecycline in the ascorbic acid and pyruvate-containing formulation, we evaluated pharmacokinetic properties of this formulation in mice following intraperitoneal administration (**Figure 3A**). The novel formulation exhibited similar half-life as tigecycline (110.3 vs. 108.9 min), indicating that the kinetics of tigecycline's elimination from the blood stream remain unchanged. No significant differences where observed in the pharmacokinetic parameters between the novel formulation and tigecycline.

Next, we evaluated the antileukemic efficacy of this novel formulation in an AML xenograft model. OCI-AML2 cells were engrafted subcutaneously in NOD/SCID mice. Following engraftment, mice were treated with saline, formulation solvent, 50 mg/kg tigecycline in saline, or 50 mg/kg novel tigecycline formulation over 11 day period. Tigecycline in saline and the novel formulation were prepared fresh twice a day. Both tigecycline and the novel formulation reduced tumor volume and weight with similar efficacy (**Figures 3B–C**).

Given that tigecycline inhibits mitochondrial translation, we next assessed the expression of mitochondrially translated proteins in the excised tumors (**Figure 3D**). Tumors treated with tigecycline and the novel formulation had similar reductions in mitochondrially translated Cox-1 and Cox-2 proteins compared to saline and formulation solvent controls, indicating that the novel formulation has similar effect on the mitochondrial function as tigecycline. Note, that the expression of nuclear-encoded Cox-4 was unchanged in all the treatment groups.

Thus, tigecycline reconstituted in ascorbic acid and pyruvate preserves its pharmacokinetic and pharmacodynamic properties, including mitochondrial translation inhibition, and maintains its antileukemic activity *in vivo*.

### The novel stabilizing formulation is well tolerated in mice

**Figure 4:**
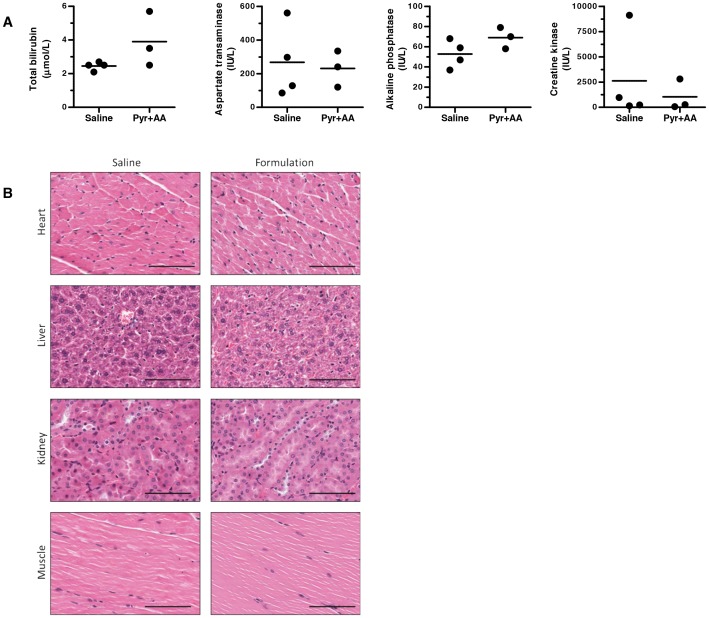
The novel ascorbic acid- and pyruvate-containing formulation displays similar tolerability in mice to saline. NOD/SCID mice (n = 3 per group) were administered saline or the novel formulation (60 mg/mL pyruvate (Pyr), 3 mg/mL ascorbic acid (AA) in saline, pH 7.0) by intraperitoneal injection 5 of 7 days over 3 weeks. At the end of the experiment, mice were sacrificed and serum and organs were collected. (**A**) Serum levels of total bilirubin, aspartate transaminase (AST) and alkaline phosphatase (ALP) were measured as indicators of liver function, while creatine kinase levels were measured as an indicator of muscle, heart or brain injury. (**B**) Heart, liver, kidney and muscle organs were sectioned and stained with hematoxylin and eosin. Representative sections from organs are shown of 1 section from 1 of 3 mice per group. Images were collected using a ScanScope XT microscope at 10× magnification. Scale bars are 100 µm.

Next, we evaluated the tolerability of the novel tigecycline formulation in mice. NOD/SCID mice were administered daily intraperitoneal injections of the novel formulation (ascorbic acid and pyruvate) or an equivalent volume of saline, 5 days per week for 3 weeks. Compared to the saline-control treated mice, no change in behaviour or weight was observed in the mice that received ascorbic acid and pyruvate. At the end of the 3 weeks of treatment, mice were sacrificed and blood was collected to assess liver chemistry and muscle function. No substantial changes in serum markers of organ injury or dysfunction were observed (**Figure 4A**). Likewise, at the time of sacrifice, heart, liver, kidney and muscle organs were harvested and stained with hematoxylin and eosin for histological evaluation. No gross or histologic changes in organ function were seen (**Figure 4B**). Thus, the novel formulation of ascorbic acid and pyruvate was well tolerated in mice for at least 3 weeks.

## Discussion

In this study, we identified a formulation (3 mg/mL ascorbic acid, 60 mg/mL pyruvate in saline, pH 7, protected from light) that improves the stability of tigecycline after reconstitution. Pending clinical evaluation of its pharmacology and safety in humans, this formulation may offer substantially more flexibility in dosing schedules and drug preparation.

The novel formulation identified contains two oxygen-reducing agents – ascorbic acid and pyruvate – that may protect tigecycline from oxidative degradation through complementary mechanisms. Ascorbic acid is a water-soluble sugar acid reported to have both antioxidant and pro-oxidant activities under different environmental contexts [23]. As a reducing agent, ascorbic acid is more readily oxidized than tigecycline. Therefore, it may protect tigecycline by blocking chain reactions that occur during tigecycline auto-oxidation. Of note, a similar reducing agent, sodium metabisulphate, has previously been proposed as an excipient in a stable parenteral tigecycline formulation in combination with EDTA [24]. However, our data indicate that ascorbic acid alone is not sufficient to fully stabilize tigecycline over several days. True antioxidants that directly neutralize oxygen radicals like hydrogen peroxide may be required to combat free radicals when ascorbic acid is depleted in solution. Sodium pyruvate, a monocarboxylate produced during glycolysis, is such an antioxidant and thus may protect tigecycline from oxidative damage through a second mechanism, complementary to the action of ascorbic acid. We also observed that the oxygen-neutralizing biocatalytic agent Oxyrase enhanced tigecycline stability in solution. However, it is more suitable for use as a biological tool than as a clinical excipient.

In our initial screen for tigecycline-stabilizing excipients, some additives previously shown to stabilize tigecycline did not have this effect as single agents at the concentrations tested. For example, sugars and sugar alcohols such as glucose and mannitol have been proposed to maintain tigecycline stability in solution for up to 6 hours following reconstitution and 18 hours following subsequent dilution [14]. However, these types of additives are primarily used to ensure tigecycline stability in its lyophilized powder form, and our study demonstrates that these excipients were incapable of stabilizing tigecycline for 24 hours after dilution in saline at neutral pH. In addition, the antioxidant chelator EDTA has been reported to stabilize tigecycline in solutions appropriate for parenteral injection as a single agent at pH 5.0 [24]. While we confirmed that this additive did indeed stabilize tigecycline in saline solution at pH 5.0 (data not shown), it did not demonstrate single-agent activity at neutral pH. Thus, our novel formulation addresses a key clinical challenge by stabilizing tigecycline in a diluted solution with a neutral pH appropriate for parenteral injection.

In this study, we identified that the degradation of tigecycline after reconstitution is accelerated by exposure to light. It is well known that tigecycline degradation is oxygen-dependant and that such measures as purging oxygen from tigecycline solutions, preparing fresh solutions and culture media or using oxygen-reactive enzymes such as Oxyrase for *in vitro* experiments, and decreasing storage temperatures can significantly improve tigecycline stability at neutral pH [15], [16], [25]. The destabilization of tigecycline in solution exposed to UV-containing light could be due to the production of free radicals from oxygen, which become major players in the oxidative degradation of the drug.

In the light-protected novel formulation, the known antibacterial and antileukemic activities of tigecycline were maintained, and comparable activity was observed whether tigecycline solutions were freshly prepared or after several days of preincubation. In addition, the novel tigecycline formulation displayed similar pharmacodynamics and pharmacokinetics as compared to tigecycline following intraperitoneal administration. Moreover, the novel tigecycline formulation preserved the antileukemic activity in a mouse model of human AML. Thus, after completing appropriate additional toxicology and pharmacology studies, this formulation could be advanced to testing in human patients, particularly in clinical contexts where more flexibility in dosing schedules, drug preparation timelines, and/or the ability to administer ambulatory infusions would be beneficial.
